# Routine Immunization Microplanning Challenges and Opportunities in Low- and Middle-Income Countries: A Mixed-Method Landscape Analysis

**DOI:** 10.3390/vaccines12121370

**Published:** 2024-12-04

**Authors:** Nicole Salisbury, Iqbal Hossain, Parysa Oskouipour, Audry Hong, Elan Ebeling, Jessica C. Shearer, Emily Grapa

**Affiliations:** 1 PATH, Seattle, WA 98121, USA; nsalisbury@path.org (N.S.); eebeling@path.org (E.E.); jess.shearer@gmail.com (J.C.S.); 2 JSI Research and Training Institute, Arlington, VA 22202, USA; iqbal_hossain@jsi.com (I.H.); parysa_oskouipour@jsi.com (P.O.)

**Keywords:** immunization, vaccine, microplanning, microplan, LMIC

## Abstract

Background: Microplanning is widely recognized as a critical tool for improving immunization coverage and equity and is considered a core component of routine immunization. However, there is limited evidence on how microplans are developed and implemented and the effectiveness of microplanning. As such, this study sought to review the existing evidence on implementation and institutionalization of microplanning; identify strategies to improve microplanning; and document evidence on new approaches to microplanning, including digitally enhanced and integrated microplanning. Methods: We employed a three-stage mixed-method approach. First, we conducted a literature review on microplanning for routine immunization. Second, we administered an online survey to gather insights into the factors that constrained and enabled microplanning in low-resource settings. Third, we conducted key informant interviews to better understand the barriers and enablers. Results: We found a paucity of published literature describing the drivers and effectiveness of microplanning and how to sustain it over time. Our review indicates that factors at both the development and implementation stages influence implementation and whether the process is sustained over time. These include the level of community engagement and health care worker ownership, access to data, the complexity of the microplanning tools, and the extent to which supervisors follow up on the plans. Conclusion: Our review indicates that microplanning is successful when health care workers and communities are engaged in the development process. While these findings highlight the benefits of a ‘bottom-up’ approach to microplanning, this may be more resource-intensive, and there remains a need for more research on the costs and benefits.

## 1. Introduction

Microplanning is widely recognized as a critical tool for improving routine immunization coverage and equity. Microplanning is an intervention used to systematically define the activities, resources, timing, and location of immunization services, particularly to reach underserved or under-immunized populations. Initially introduced to immunization programs as a component of polio eradication activities, microplanning was incorporated into global guidance for routine immunization with the introduction of the Reaching Every District (RED) approach in 2002 [1].

The RED approach was developed by WHO, UNICEF, Gavi, and other technical partners with the aim of improving immunization coverage in poorer-performing health districts and facilities [1]. The RED approach outlines operational components that immunization program staff can follow to improve immunization systems. Key among these components is the development and implementation of a comprehensive microplan using local data to map the target population, plan appropriate activities to reach the target population, monitor the data, and work with the community to track children who have missed routine immunization doses.

Microplanning is a core component of routine immunization and has been incorporated into several iterations of global guidelines [2,3]. However, there is limited evidence on whether and how these guidelines are followed in practice, particularly in resource-constrained settings. Most low- and middle-income countries (LMICs) have adopted microplanning through the RED approach, but there is a lack of rigorous studies measuring its effectiveness for increasing routine immunization coverage [4]. Moreover, there is a lack of evidence about whether and how microplanning processes have been institutionalized (or routinized) over time, with microplans being updated at regular intervals and implementation being adjusted as needed. Anecdotal evidence suggests that even where developed, microplans are still suboptimally implemented and institutionalized in routine immunization programs.

MOMENTUM Routine Immunization Transformation and Equity (hereafter referred to as “the project”) applies best practices and explores innovations to address entrenched obstacles in immunization and increase equitable immunization coverage. The project is USAID’s flagship technical assistance mechanism for immunization in more than 20 countries and seeks to contribute to the global evidence base on interventions to improve routine immunization coverage. The project led this study to the following:

1. Review the existing evidence on the implementation and institutionalization of microplanning for routine immunization;
2. Identify strategies to improve microplanning for routine immunization;
3. Document evidence on new approaches to microplanning which could be applicable for routine immunization contexts, including digitally enhanced microplanning and integrated microplanning.

## 2. Materials and Methods

### 2.1. Study Design

The project employed a three-stage mixed-method approach to investigate factors affecting microplanning implementation and institutionalization in LMICs. First, the team conducted a literature review of peer-reviewed and gray literature on microplanning for routine immunization. Second, the team administered an online survey designed to gather insights into the drivers of microplanning from national and subnational immunization program staff and technical partners. Third, the team conducted in-depth key informant interviews with a subset of stakeholders identified in the survey to better understand the enablers and barriers to successful implementation and institutionalization of microplanning. The project employed the Consolidated Framework for Implementation Research (CFIR) throughout the research process to guide the analysis [5].

#### 2.1.1. Literature Review

The team searched three databases (PubMed, CABO Web of Science, and Proquest) for relevant articles using the following search strings: (“vaccine” OR “immunization” OR “immunization program” OR “routine immunization”) AND (“microplan” OR “micro plan”). The team reviewed only English language articles and did not restrict articles by date of publication. To augment the search, the team reviewed references of relevant articles to identify additional sources and performed a Google search to identify relevant gray literature.

**Figure 1:**
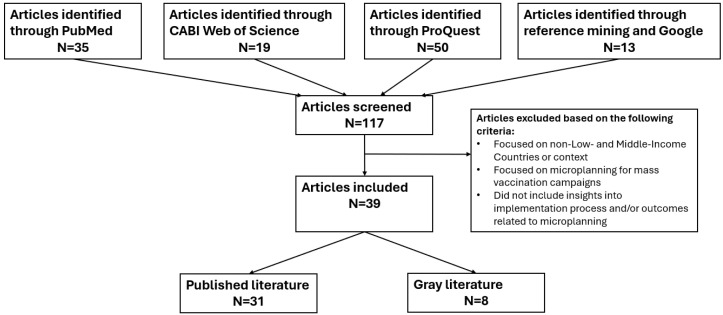
Flow chart diagram of article selection.

The literature review was conducted from June to July 2023. Initial searches yielded a total of 117 results. After screening based on exclusion criteria and using a double review on a random selection of abstracts to minimize exclusion bias, the team included 39 articles, reports, or briefs for full-text review (Figure 1). Of these sources, 27 described experiences with microplanning in Africa, 10 in Asia, and 2 in Latin America and the Caribbean. Articles were coded using Excel according to the six domains of the CFIR framework (innovation, outer setting, inner setting, individuals, implementation process, and implementation outcomes) to distill the factors that constrained and enabled microplanning implementation. 

#### 2.1.2. Online Survey

To better understand the factors that influence microplanning implementation, the project developed and administered an 11-question online survey targeting stakeholders with experience implementing microplanning in LMICs. The survey tool (Google Forms) included three sections based on the crucial stages of the microplanning process: development, implementation, and institutionalization. The survey was disseminated from June to August 2023 via three online forums (Boost Community Network, Zero-Dose Community of Practice, and TechNet-21 Forum) whose reach includes immunization program managers from LMICs at national and subnational levels. Respondents were given the option to provide their name and contact information if they were open to being interviewed but were not required to do so. Survey results were exported to Excel. Summary statistics were analyzed, and open-ended questions were coded according to the CFIR framework. Survey results were primarily used to (i) identify key respondents for interviews and (ii) inform the development of topic areas for the interview guides used in the third step of the research process.

#### 2.1.3. Key Informant Interviews

As a third step, the team conducted in-depth semi-structured key informant interviews with stakeholders to learn more about their experiences with microplanning implementation and institutionalization. From the countries represented in online survey responses, the project employed a purposive sampling approach to select a subset of countries with varying levels of partner support for routine immunization to reflect different resource contexts within immunization programs. The key informants included technical partners and national or subnational immunization program staff.

Drawing from themes gathered in the online survey responses, the project developed a semi-structured interview guide with the aim of probing further into the factors constraining and enabling implementation and institutionalization of routine immunization microplanning. Interviews were conducted remotely in English or French by project staff and lasted up to one hour. Interviews were recorded with participant consent. Interview transcripts were uploaded to a password-protected folder and coded in Dedoose according to the six CFIR domains.

### 2.2. Root Cause Analysis

To triangulate and synthesize findings from across the research steps, the project conducted a root cause analysis (RCA) using data collected from the literature review, online survey, and key informant interviews. Due to the paucity of evidence on microplan implementation in the published literature, RCA drew primarily from key informant interview data, triangulated where possible with insights from the literature review and online survey. RCA is an analytic process to identify underlying causes of key process challenges, often by developing a visual diagram that elucidates the causal chain between the challenge and underlying root cause(s) [6]. Project staff who led the data collection and analysis for each research step participated in an iterative RCA process. Coded data on the constraints and enablers of microplanning implementation from all data sources were synthesized and revised iteratively upon review by the technical team. Through the iterative RCA process, the team identified root causes affecting the microplanning process, visualized the relationships between root causes, and organized the root causes based on the following: (1) whether they were related to the development or implementation of microplans and (2) whether they were enablers or barriers of these processes. 

### 2.3. Ethics

IRB exemption was obtained from PATH IRB, as this was determined to be research on non-human subjects. The online survey was voluntary, and participants were informed of the survey’s purpose and how the data they provided would be used. Survey respondents provided informed consent and were provided the option to include their contact information if they consented to participate in follow-up interviews. Interview participants provided verbal consent to participate. All study data that contained identifiable information were stored in a password-protected folder only accessible to the study team to ensure the privacy and confidentiality of participants. 

## 3. Results

### 3.1. Description of Sample

**Figure 2:**
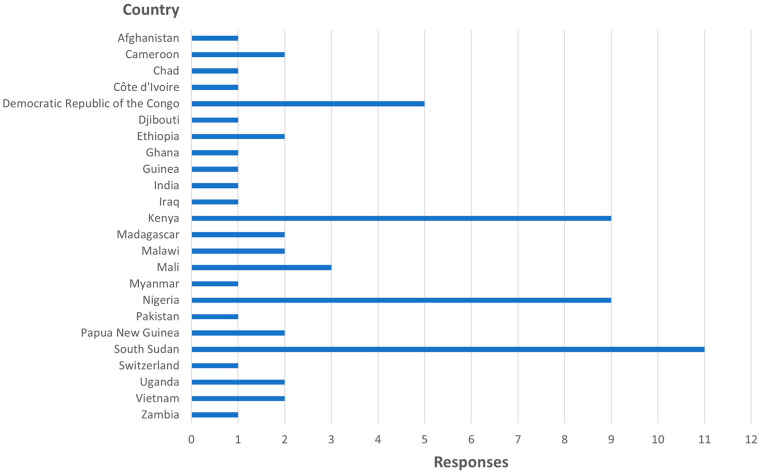
Survey respondents by country.

The online survey was completed by 63 individuals from 24 countries (Figure 2). The survey included respondents from all six WHO regions (Africa, Americas, Eastern Mediterranean, Europe, Southeast Asia, Western Pacific), with the majority of responses from the Africa region. The team conducted 14 interviews with individuals from seven countries who are involved in microplanning implementation, including eight technical partners, two national-level routine immunization program staff, and four subnational immunization program staff (Table 1).

### 3.2. Key Findings

Through the RCA, the project identified two broad categories of factors which constrain and enable the implementation and institutionalization of microplanning processes in routine immunization programs: (1) the process of developing microplans and (2) the implementation of microplans. In addition, digitally enhanced microplanning and integrated approaches to microplanning emerged as two cross-cutting approaches to strengthen both microplanning development and implementation.

#### 3.2.1. Microplan Development

##### Tools and Templates

RED global guidelines provide tools and guidance to support microplan development at the health facility level. The guidelines begin with a table to analyze data on target populations and immunization coverage in order to categorize and prioritize challenges related to access and utilization. This is used to inform other tools, including an operational map, a session plan, a quarterly work plan, a stock record, a drop-out tracking system, and a monitoring chart. Microplanning tools and templates provide guidance on the information required to develop high-quality microplans. However, according to the literature and key informants, users often find the tools to be complicated. One key informant summarized the following:

“Tools are not friendly for the lower levels. They need simplification for health care workers (HCWs); making tools friendly for HCWs is important.”

(Technical partner, Ethiopia)

The literature and key informants indicated that challenges stem from the length and number of tools and templates, the detailed information required to populate them, and the Excel-based format of some templates.

“Challenges related to microplanning were related to the templates. They are Excel-based, and for those staff who were not used to Excel, it was difficult for them to use. Nothing was automated on the Excel templates, they were just using it as a table. So it was not as useful as it has the power to be.”

(Technical partner, Ethiopia)

Challenges using microplanning tools can have consequences for the quality of microplans. For instance, a qualitative assessment of the microplanning process in two districts in Uganda found that the complex and bulky nature of the microplanning tool contributed to only 57 percent of health facilities having an updated microplan [7].

To address this challenge, the team found evidence in the literature and in key informant interviews of adaptations made to guidelines, tools, and templates to enhance their user-friendliness and contextual relevance. For example, in Tanzania, microplanning tools were adapted and integrated with other council-level planning tools [8]. In Uganda, one key informant described how microplanning tools and processes have been simplified over time to minimize health worker burden.

##### Access to High Quality and Timely Data

Microplanning requires accurate, timely data on target populations and catchment areas. However, accessing high-quality denominator data was a commonly cited challenge. According to key informants,

“During microplanning, the problem is the denominator. Most places use the census for population estimations, but the census is old. And anyway, there is massive population movement. So having the right denominator for planning is one factor that enables or hinders microplanning.”

(Technical partner, Ethiopia)

As another key informant noted, those developing the microplan may be compelled to use the often outdated and inaccurate census population estimates because they are the official data used by the health system. This may lead to inaccurate estimates of vaccines and other supplies needed, poor quality coverage data, or failing to reach underserved populations [7].

Local enumeration activities can provide up-to-date, high-quality data on target populations, but this process can be time- and resource-intensive. For example, a study in Kaduna State, Nigeria, found that the number of children <1 year of age identified through microplanning household enumeration was 84 percent higher than the estimates based on census data [9]. The authors concluded that periodic household-based microplanning is useful for more accurate denominators but noted that the process required significant manpower and resources from many partners and government agencies. 

Digital tools, especially geospatial technologies, are another potential strategy to map target populations for more accurate estimates. However, some key informants cautioned that digital tools may support the microplanning process but should not replace community engagement. Digital tools, without community insights, cannot accurately capture migration patterns, community movement, or perspectives on the timing and location of outreach services (see Section Digitally Enhanced Microplanning).

##### Health Worker Capacity

Evidence from the literature and key informants pointed to a lack of training, supportive supervision, and mentoring on microplanning as a key challenge to the development and implementation of high-quality microplans. Key informants noted that health care workers (HCWs) often have limited experience in immunization, and training in microplanning is variable or insufficient for HCWs to effectively use the microplanning tools.

“The majority of staff are new employees and have no immunization background and no experience in microplanning, and [there are] no operational-level trainings for the majority of facilities. We need to build the capacity of our health care workers… Part of the job contract should actually be development and implementation of microplans.”

(Subnational immunization program staff, Kenya)

Additionally, high staff turnover was commonly mentioned as a challenge that creates microplanning knowledge gaps in facilities. Key informants in Uganda, DRC, and Vietnam provided examples of capacity strengthening activities, such as cascade training, peer exchanges, and mentoring activities implemented by donor-funded projects. However, the sustainability of many of these programs was cited as an ongoing challenge as they remained dependent on partner support and/or donor funding. Engaging HCWs and health facility managers in the process of developing plans and thoughtful capacity strengthening activities was reported to increase motivation to actually implement the activities in the plan (see Section Health Worker Capacity).

##### Community Engagement

Over time, approaches to microplanning have evolved. In the early days of RED implementation, microplans were often developed through a top-down approach, where ministries of health and technical partners convened workshops for facility and district-level immunization program managers. As the focus of immunization programming has shifted toward reaching missed communities and zero-dose children, a new approach to microplanning, sometimes referred to as “bottom-up microplanning”, has emerged. The bottom-up approach starts at the health facility level and emphasizes community engagement in mapping target populations and identifying barriers to immunization. This is consistent with the increased emphasis on community engagement in the microplanning process in the 2017 RED guide from the WHO Regional Office for Africa [3].

The engagement of community members and other non-traditional stakeholders was broadly acknowledged as being critical to the development of high-quality microplans. Key informants reported that the participation of community stakeholders resulted in microplans that were more reflective of local needs and priorities, particularly in the location and timing of immunization sessions. In addition, the participation of local stakeholders encouraged greater local buy-in into microplanning implementation, including local efforts to mobilize resources to support immunization planning. However, engaging communities in the microplanning process requires additional resources, such as reimbursement for transportation costs, which immunization programs may not have.

According to a key informant in Nigeria,

“Successful microplans need to involve community members and community-based organizations to help with outreach. Have people come to a selected place, whether it’s the health facility or the leader’s home. However, you need transportation, preparation, and meals–resources in general–to involve the community.”

(Technical partner, Nigeria)

#### 3.2.2. Microplan Implementation

##### Health Worker Ownership

Cultivating ownership was found to be an important factor for motivating health workers to develop and implement microplanning. For example, JSI supported microplanning implementation in 25 districts in Uganda from 2014 to 2019 and found that HCWs experienced a higher degree of ownership over the microplans when they were engaged in the process of developing the plans [10]. Conversely, Mafigiri et al. found that HCWs in Uganda who were not involved in the microplan development process did not understand how to implement them, despite recognizing the value of microplanning to improve immunization services [7].

“Being committed to carry out a plan that people themselves developed wound up being really important. And we also found that it was important to involve the clinic managers in some aspects of the microplanning process because ultimately, they could control where some of the resources went, and played a really important role in implementation.”

(Global technical partner)

##### Supportive Supervision, Mentorship, and Accountability

Other factors that key informants indicated enhance health worker ownership, and motivation for implementing microplans were supportive supervision and mentorship, coupled with monitoring and accountability mechanisms from higher levels of the health system. One best practice cited by key informants for monitoring microplanning was creating a WhatsApp group to send reminders to immunization focal persons at the subnational level. The WhatsApp groups provided a venue for higher level authorities to encourage microplan development and use. Conversely, a lack of follow-up from higher levels was mentioned as a constraint, particularly for institutionalizing microplanning.

“Microplanning has not been advocated very well… it still needs follow up from the high levels to be institutionalized. It is not well aligned with existing mechanisms like district-based plans. There are competing tools that sometimes the higher levels use instead of microplans.”

(Technical partner, Ethiopia)

##### Resources to Support Implementation

Survey respondents and key informants both indicated that the implementation of microplans has been constrained by a consistent shortfall in operational funds for immunization programs at the subnational level to support planned activities, such as outreach, to provide services to unreached populations and zero-dose children. This challenge is not specific to microplanning but reflects larger resource constraints for immunization in many countries. 

To mitigate this challenge, the 2017 RED guide from the WHO Regional Office for Africa suggests preparing budgets to carefully balance the resources needed with the resources available to implement strategies to reach zero-dose children. Key informants also emphasized the importance of advocacy to generate political will for making operational funds and other resources available for the implementation of the activities outlined in microplans. Key informants suggested addressing the shortfalls of government funding by mobilizing other sources (such as donors, the community, or the private sector) and by prioritizing critical activities to reach zero-dose children. For instance, in Tanzania, bottom-up microplanning was performed in the context of the annual Comprehensive Council Health Plan (CCHIP) process, which was documented as a promising approach for aligning the funds required for outreach and vaccine distribution with the available budget [11]. 

Several key informants mentioned a lack of funds to even print the final microplan, limiting HCWs’ ability to implement the plans:

“The health facilities were compelled to send their microplans to the district level without any copy left for their own reference. They were not sent back. The facilities that had developed their microplans and that were committed to carrying them out, that knew what they were going to do with them, it left them without those microplans.”

(Technical partner, Uganda)

#### 3.2.3. Adaptations to Microplanning

This study aimed to learn more about what adaptations to traditional microplanning may be taking place and whether and how those adaptations may be strengthening development, implementation, or institutionalization of microplans. Throughout the course of the study, two cross-cutting adaptations in particular came up during both the literature review and key informant interviews.

##### Digitally Enhanced Microplanning

Digitally enhanced microplanning includes the utilization of tools such as digital mapping, mobile applications, online training forums, and electronic registries. These tools are used to help map catchment areas, identify target estimates, train health care workers, develop budgets, and monitor vaccine status. Geo-enabled microplanning was the most commonly cited form of digitally enhanced microplanning in the literature review. Geo-enabled microplanning entails using geospatial data to develop digital maps that capture information like the location of settlements, infrastructure, roads, and geographic terrain [12]. 

Key informants reaffirmed the benefits of digitally enhanced microplanning, citing that such activities result in improved mapping, better estimation of target populations, optimization of staffing assignments, and more efficient resource allocation. However, key informants were also clear in outlining barriers to widespread use of digitally enhanced microplanning, which can include poor technological infrastructure and internet connectivity, limited information technology (IT) capacity of HCWs and other staff, the high costs of making updates to microplans, and continued reliance on technical assistance from partners. In Vietnam, the national immunization information system uses digitized microplanning forms and processes. However, key informants described scenarios at the subnational level wherein health care workers lack the IT skills necessary to make use of these tools. As a result, some HCWs complete paper-based forms for data entry, which must be later manually reconciled with the digitized national immunization system. Despite these occasional incongruencies, the key informants still perceived digitally enhanced microplanning to contribute to more accurate microplans that reduce wastage of vaccines and other materials. Critically, key informants also made note that digital tools should ideally serve as enhancements to the microplanning process and should not replace the role of community engagement in setting priorities and identifying missed communities. 

##### Integrated Microplanning

Integrated microplanning presents an appealing prospect in resource-constrained settings because, theoretically, it has the potential to reduce costs by combining multiple necessary interventions into one microplanning process. The peer-reviewed literature review yielded only two articles that described an integrated approach to microplanning. In Mongolia, microplans were developed for immunization and maternal and child health (MCH) services [13]. While the study did conclude that microplanning was well-suited to other interventions beyond immunization, it did not provide details of any specific barriers or enablers to taking an integrated approach or any measure of the effectiveness of the approach. A study in Uganda reported on efforts to integrate maternal health, nutrition, and child health into a single microplanning tool but cautioned that doing so could make the process more cumbersome and, therefore, increase the workload of HCWs who are already stretched thin [7].

Key informants expressed a growing readiness to adapt integrated approaches to microplanning, particularly in the context of efforts to strengthen primary health care (PHC). PHC is widely considered as a critical pathway to universal health coverage, and immunization is typically identified as a necessary component of robust PHC systems. Given this relationship, there is interest in identifying opportunities for better integration between immunization and other PHC services from program planning to service delivery since it may represent a more holistic use of resources than each health program operating in isolation. This appeared to be the case in Nigeria, where key informants reported increased emphasis on working toward integrated microplanning for PHC. In Uganda, one key informant described how this type of integrated microplanning manifests between immunization and nutrition services, such as Vitamin A supplementation for children aged one year old. In others, the scope is wider, comprising broader MCH services and family planning. 

Limited human and financial resources were commonly reported by key informants as barriers to integration, particularly in the implementation phase. A key informant in Uganda reported that identifying zero-dose and missed communities for immunization microplanning provides an entry point to offer additional services (including MCH and nutrition services), but implementation requires additional resources. They noted,

“…we are grappling with finding additional resources in delivering the integrated packages, because when I deliver an integrated package I’m going to need more health workers, I’ll need additional transport. I need additional vaccine carriers. So you need more resources, the resources almost double because of the integrated package.”

(Technical partner, Uganda)

Another global technical partner noted in their interview that, while many perceived microplanning to be a window of opportunity for more holistic planning for immunization as part of PHC, this approach was not presently being leveraged to the extent it could be. The key informant attributed this in part to siloed funding sources and distinct measurement and reporting requirements by health area. 

## 4. Discussion

The project found very little published and gray literature on microplanning in the context of routine immunization programs. Where microplanning was discussed, it was often as one component of the broader RED approach to improve immunization coverage and equity, without mention of factors that specifically constrained or enabled the microplanning process. Therefore, the team relied primarily on key informant interviews to explore the enablers and barriers to microplan development and implementation. Moreover, the review of the literature confirmed a lack of evidence on whether, how, and why microplanning contributes to immunization outcomes. This is consistent with a recently published evidence review of pro-equity interventions, which found microplanning to be a promising intervention, but noted the lack of rigorous analysis of the effectiveness of microplanning on immunization outcomes [14].

Many implementation constraints identified, such as a lack of HCW ownership, accountability, and operational resources, are not specific to microplanning and instead more broadly reflect barriers to implementation of routine immunization programming [15]. As such, the sub-optimal implementation of microplans should not necessarily be interpreted as shortcomings of microplanning alone but rather as a reflection of broader weaknesses in routine immunization systems.

This review indicates that microplanning is most successful when HCWs are engaged in the development process, thereby increasing their ownership over the process and the likelihood of the plans being implemented. Likewise, community participation was found to contribute to the enhanced recognition of the barriers missed communities face in accessing immunization services, and to better inform the development of remedial strategies. While these findings highlight the benefits of a “bottom-up” approach to microplanning, key informants cautioned that it is resource-intensive, and there remains a need for robust research and documentation on the costs and benefits of this approach. 

A concerted and ongoing effort is required for strengthening the capacity of those HCWs responsible for developing, updating, and improving the quality of microplans. The tools and templates for developing a microplan were widely considered to be complicated, and key informants highlighted a need to simplify the microplan templates and guidance. Without proficiency among HCWs and district managers in using the tools and templates, key informants reported that the resulting microplans did not well reflect community needs or the most effective strategies to reach zero-dose children and missed communities. However, it was also emphasized that identifying missed communities and zero-dose children is inherently complex and resource-intensive, so comprehensive tools are necessary to support this process. Therefore, it is important to strike a careful balance between an appropriate level of simplification while also providing ample information and level of detail actually required to identify zero-dose children, understand their barriers to immunization, and reach those communities with high-quality immunization services. 

The project found a growing emphasis on digitally enhanced microplanning, especially the use of digital tools and applications to map catchment areas, identify high-risk populations, and estimate populations. Although there is some evidence to support the effectiveness of this approach in arriving at more accurate estimates, they must not be viewed as a replacement for community engagement, as communities may not accept figures if they are not engaged in the process of developing those estimates. Furthermore, digital tools may produce estimates that are inconsistent with officially sanctioned figures that are typically generated using census data and used by different programs across the health system. HCWs must also be sufficiently capacitated to use the digital tools. As such, in order to use digital tools, there is a need for higher level policy decisions. Finally, while digitally enhanced microplanning can address some of the challenges identified through this review, it cannot address all and may in fact exacerbate some without careful evaluation of the costs and benefits. 

Due to the widespread uptake of and adherence to the normative framework laid out through the RED guidance starting over 20 years ago, microplanning is broadly acknowledged to be part and parcel of immunization programming for both campaigns and routine immunization. Ministries of Health, and country Expanded Programs on Immunization (EPIs), have reinforced this to varying degrees by requiring that health districts and facilities engage in microplanning. The sub-optimal implementation of those plans is a direct consequence of insufficient resources to both develop microplans, and then to implement the activities therein. 

## 5. Conclusions

In conclusion, the project found that the level of implementation and institutionalization of microplanning for routine immunization was highly variable from setting to setting (between and within countries) and, in many, still highly dependent on partner resources. It is, therefore, important to advocate for increased government recognition of the purpose and value of immunization in improving child health, as well as for adequate resources. Such government endorsement will also contribute to a virtuous cycle, whereby microplans are developed and implemented and the process is institutionalized, as an essential component of achieving equity in immunization.

## 6. Limitations

This study has several limitations that should be acknowledged. First, although a systematic literature search was conducted, some relevant articles may have been overlooked, particularly those found in gray or unpublished sources or not published in English. Second, the identified literature had limited details about the drivers of microplanning, and microplanning was often discussed as one component of RED/REC guidance and programming, making it difficult to disentangle the drivers of microplanning from those affecting routine immunization more broadly. Third, due to the limited evidence in the literature focused specifically on microplan development and implementation, the project relied heavily on key informant interviews. Due to time and resource constraints, the team interviewed a limited number of key informants, which was insufficient to achieve theoretical saturation. Although the team selected key informants from nine countries who offered valuable insights, these perspectives may not fully capture the range of experiences across countries and do not include the perspectives of community members. Despite these limitations, this review offers practical insights into the factors that facilitate or hinder effective microplanning in varied contexts, which can help to inform stakeholders involved in designing and implementing microplanning.

## Figures and Tables

**Table 1:** Interviews conducted by country and stakeholder type.

Country	Number of Interviews	Types of Stakeholders Interviewed
DRC	3	Technical partner, National level EPI staff, District health staff
Ethiopia	2	Technical partner
Kenya	2	District health staff
Nigeria	2	Technical partner
Uganda	2	National level EPI staff, District health staff
USA	1	Technical partner
Vietnam	2	Technical partner
Total	14	

## Data Availability

The data presented in this study are available upon request from the corresponding author.
